# Long-term safety and efficacy of opicapone in Japanese Parkinson’s patients with motor fluctuations

**DOI:** 10.1007/s00702-021-02315-1

**Published:** 2021-02-25

**Authors:** Atsushi Takeda, Ryosuke Takahashi, Yoshio Tsuboi, Masahiro Nomoto, Tetsuya Maeda, Akihisa Nishimura, Kazuo Yoshida, Nobutaka Hattori

**Affiliations:** 1 National Hospital Organization, Sendai-Nishitaga Hospital, 2-11-11 Kagitorihoncho, Taihaku-ku, Sendai, 982-8555 Japan; 2 Department of Cognitive and Motor Aging, Tohoku University, Graduate School of Medicine, Sendai, Japan; 3 Department of Neurology, Kyoto University Graduate School of Medicine, Kyoto, Japan; 4 Department of Neurology, Fukuoka University Hospital, Fukuoka, Japan; 5 Department of Neurology and Clinical Pharmacology, Ehime University Graduate School of Medicine, Ehime, Japan; 6 Department of Neurology, Saiseikai Imabari Hospital, Ehime, Japan; 7 Division of Neurology and Gerontology, Department of Internal Medicine, School of Medicine, Iwate Medical University, Iwate, Japan; 8 Department of Clinical Development, Ono Pharmaceutical Co., Ltd., Osaka, Japan; 9 Department of Neurology, Juntendo University Graduate School of Medicine, Tokyo, Japan

**Keywords:** Japanese, Open-label, Opicapone, Parkinson’s disease

## Abstract

The double-blind part of the COMFORT-PD (COMt-inhibitor Findings from Opicapone Repeated Treatment for Parkinson’s Disease) study in Japanese levodopa-treated patients with Parkinson’s disease and motor fluctuations found that both opicapone 25 and 50 mg were significantly more effective than placebo. This 52-week open-label extension study evaluated the long-term safety and efficacy of opicapone 50 mg tablets in patients who completed the double-blind part of the COMFORT-PD study. Safety was monitored via adverse events, laboratory testing, and physical, cardiovascular and neurological examinations. Efficacy was primarily assessed by change in OFF-time. Secondary efficacy measures included: ON-time, percentage of OFF/ON-time responders, other outcomes from the double-blind part. 391/437 patients were transferred to the open-label extension period and included in the safety analysis set (full analysis set, *n* = 387; open-label completers, *n* = 316). Adverse events were frequently reported (*n* = 338, 86.4%), but < 50% were considered drug-related (39.9%) and few were considered serious (2.6%) or led to discontinuation (2.8%). Decreased OFF-time was consistently observed over the open-label period regardless of initial randomization. Change [LSM (SE)] in OFF-time from the open-label baseline to the last visit showed a persistent effect in patients initially randomized to opicapone 25 mg [− 0.37 (0.20) h, *P* = 0.0689] and opicapone 50 mg [− 0.07 (0.21) h, *P* = 0.6913] whereas opicapone 50 mg led to a statistically significant reduction in the previous placebo group [− 1.26 (0.19) h, *P* < 0.05]. Once-daily opicapone 50 mg was generally well tolerated and consistently reduced OFF-time over 52 weeks in Japanese levodopa-treated patients with motor fluctuations.

*Trial registration* JapicCTI-153112; date of registration: December 25, 2015.

**Supplementary Information:**

The online version contains supplementary material available at 10.1007/s00702-021-02315-1.

## Introduction

Inhibition of catechol-O-methyltransferase (COMT) is an established strategy for treating end-of-dose motor fluctuations (wearing-off) in patients with Parkinson’s disease (PD) treated with levodopa (L-dopa) and a DOPA decarboxylase inhibitor (DCI) (Fox et al. 2018). Opicapone (BIAL, Portela & Ca, S.A.) is a novel, third-generation COMT inhibitor that provides sustained COMT inhibition making it suitable for once-daily administration, which has the potential to enhance convenience and adherence associated with long-term dosing (Farrell et al. 2013; Rocha et al. 2013; Sabbatini et al. 2014). Previous placebo-controlled, randomized clinical trials in non-Japanese populations have demonstrated that opicapone 50 mg capsules were generally well tolerated and significantly reduced OFF-time compared with placebo (Ferreira et al. 2016; Lees et al. 2017). Further, the pooled analysis of associated long-term extension studies conducted for periods of 1 year demonstrated that opicapone 25–50 mg capsules led to a sustained reduction in OFF-time without additionally increasing the frequency of dyskinesia (Ferreira et al. 2019).

Although previous studies have confirmed that the pharmacokinetic and pharmacodynamic profiles of opicapone are similar in Japanese and non-Japanese populations (Falcao et al. 2016), there has been a lack of studies on the clinical efficacy and safety of opicapone in Japanese patients. Prior to this study, we conducted a double-blind, randomized, placebo-controlled study with opicapone tablets developed by Ono Pharmaceutical Co. Ltd. (Osaka, Japan) to evaluate the efficacy and safety of opicapone 25 mg and 50 mg tablets versus placebo in Japanese patients with PD and motor fluctuations despite treatment with an L-dopa and DCI combination (Takeda et al. 2021). Results of this double-blind study found that, compared with placebo, both opicapone 25 mg and 50 mg tablets were associated with statistically significant reductions in OFF-time as well as improvements in other endpoints, including the percentage of ON-time responders and changes in total ON-time/ON-time without troublesome dyskinesia.

This open-label extension of the abovementioned double-blind study was designed to investigate the safety and efficacy of long-term treatment with once-daily opicapone 50 mg tablets in Japanese patients with PD and motor fluctuations.

## Methods

### Study design and patients

This is a 52-week open-label study implemented after the randomized, double-blind, placebo-controlled study to evaluate the safety and efficacy of long-term extension treatment with once-daily opicapone tablets at a fixed dose of 50 mg (Supplementary Fig. 1).

Methods for the randomized double-blind, placebo-controlled trial that preceded this open-label extension study have been reported elsewhere. In brief, eligible patients had a clinical diagnosis of PD [UKPDS Brain Bank Clinical Diagnostic Criteria (Hughes et al. 1992), Hoehn-Yahr stage (Goetz et al. 2004) 1–3 at ON stage] for ≥ 3 years with a ≥ 1-year history of clinical improvement with L-dopa plus DCI therapy, and wearing-off motor fluctuations (mean total awake OFF-time ≥ 1.5 h, excluding morning akinesia) for ≥ 4 weeks before the screening period. Patients also had to have received a stable optimized regimen of 3–8 daily doses of L-dopa plus DCI therapy and other PD medications for ≥ 4 weeks before screening.

Randomized patients from the double-blind treatment period who were able and willing to continue to the open-label period were transferred via the transfer period after data obtained during the double-blind period was locked. During the transfer period, patients were maintained on a stable regimen of opicapone or placebo, L-dopa and DCI as had been administered at the end of the double-blind treatment period. During the open-label period, all patients received opicapone 50 mg film-coated tablets once daily at bedtime ≥ 1 h after the last administration of L-dopa and DCI. The daily dose or intake frequency of L-dopa and DCI could be increased or decreased if considered necessary for symptom control. Further, concomitant antiparkinsonian medications were permitted with flexible dose, with the exception of entacapone, which was not permitted as part of the exclusion criteria. However, initiation of any antiparkinsonian medications was not permitted. As a result, the conditions of medication use were designed to simulate those of real-world conditions as closely as possible while allowing assessment of a fixed dose of opicapone. A post-treatment observation period was included after the last dose of the open-label period to confirm the safety of patients.

### Assessments

Patients enrolled in the open-label period were assessed at the time point immediately after completion of the 4-week transfer period and at multiple intervals (generally every 4 weeks) from the open-label baseline to Week 52 (or the last visit for patients who discontinued early) via the safety and efficacy variables used during the double-blind period. Safety was primarily assessed for opicapone over 52 weeks in an open-label manner using the same assessments for adverse events, laboratory test, as well as physical, cardiovascular and neurological examinations used in the double-blind period. Suicide risk continued to be assessed via the Columbia Suicide Severity Rating Scale (C-SSRS). During the open-label period, adverse events were classified using MedDRA Version 20.1 (Japanese version) in the same manner as in the double-blind period according to severity and causal relationship to study medications with adverse events classified as at least possibly related to the study medication considered as drug-related adverse events.

Efficacy was primarily assessed by the change in OFF-time based on patient symptom diary from the double-blind baseline and open-label baseline to Week 52.

Secondary efficacy variables used in the double-blind period continued to be assessed during the open-label period. These included change in ON-time, the percentage of OFF- and ON-time responders, defined as patients whose OFF-/ON-time was reduced or increased by 60 min or more from the baseline. Other secondary efficacy variables were the absolute value and change in UPDRS items (Fahn et al. 1987), Modified Hoehn and Yahr Staging at ON stage (Goetz et al. 2004), Schwab and England ADL Scale at ON and OFF stages (Schwab and England 1969), Clinician and Patient Global Impression of Change (CGI-C and PGI-C) (Guy 1976), and the 39-item Parkinson’s Disease Questionnaire (PDQ-39) (Peto et al. 1998).

### Statistical analysis

For the open-label period, statistical analyses were performed after data lock following study completion. Statistical analyses were performed on the full analysis set, consisting of patients with ≥ 1 efficacy evaluation of the primary variable after administration in the open-label period, and the safety analysis set, which included patients who received ≥ 1 dose of opicapone in the open-label period. The primary efficacy variable (change in OFF-time from open-label baseline to the last visit) was compared using an analysis of covariance with treatment group as a factor and baseline OFF-time at the double-blind period as a covariate. Least squares means (LSM) and corresponding standard errors (SE) were calculated. The Last Observation Carried Forward (LOCF) method was applied to the handling of missing data. For secondary efficacy endpoints and safety assessments, summary statistics of continuous variables and frequency distributions of ordinal scale variables were calculated. Safety analyses were performed using the safety analysis set. SAS^®^ software (versions 9.3 and 9.4) was used for all statistical analyses.

## Results

### Patient disposition and baseline characteristics

In total, 391 of 437 patients were transferred to the open-label period after completion of the double-blind period and assessed as the safety analysis set. Of these patients, 387 patients were included in the full analysis set and 316 patients completed the open-label period. Of the 75 patients who withdrew from the study, 38 (50.7%) patients withdrew due to patient request, 26 (34.7%) patients withdrew due to adverse events, and 11 (14.7%) patients withdrew due to other reasons (Supplementary Fig. 2). Patients were highly compliant with the fixed-dose schedule of opicapone used during the open-label period. The mean compliance rate was 97.1% and 376 of 391 (96.2%) patients had compliance 80% or more.

### Efficacy

**Fig. 1:**
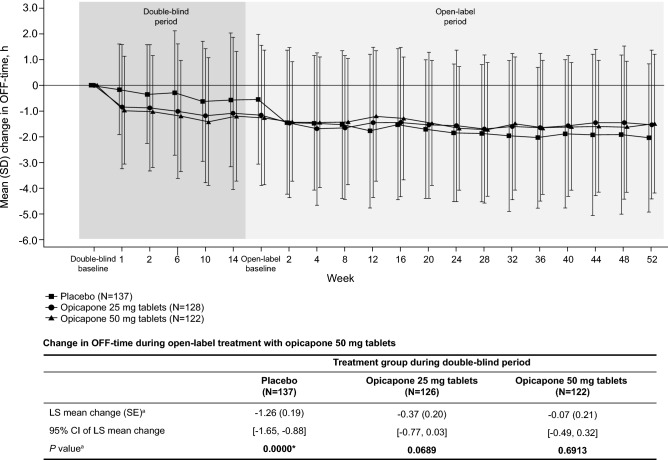
Change in OFF-time over double-blind and open-label period (graph) and LS mean (SE) changes in OFF-time from open-label baseline to last visit (Table). ^a^Estimated using an analysis of covariance with treatment group as a factor and baseline value at the double-blind period as a covariate. The Last Observation Carried Forward (LOCF) method was applied to the handling of missing data. *P < 0.05 represents significant change from open-label baseline. *CI* confidence interval, *DB* double-blind period, *NS* not significant, *LS* least squares, *SE* standard error

The change in OFF-time from baseline of the double-blind period through to the last visit of the open-label period for patients initially randomized to placebo, opicapone 25 mg tablets, and opicapone 50 mg tablets during the double-blind period is shown in Fig. 1. During the open-label period, the overall mean (SD) change in OFF-time from the double-blind period baseline through to Week 52 varied between − 1.79 (2.75) and − 1.43 (2.78) h. In addition, the LS mean (SE) change in OFF-time from the open-label baseline to the last visit for patients initially randomized to placebo, opicapone 25 mg and opicapone 50 mg is shown in the table accompanying Fig. 1. These results demonstrate that the OFF-time at the last visit from the open-label baseline showed a persistent effect in patients initially randomized to opicapone 25 mg [LS mean (SE) change − 0.37 (0.20) h] and opicapone 50 mg [− 0.07 (0.21) h] whereas switching from placebo to opicapone 50 mg led to a statistically significant reduction in OFF-time in the placebo group [− 1.26 (0.19) h, *P* < 0.05]. Similarly, the change in ON-time was statistically significant (*P* < 0.05, data not shown) in patients initially randomized to the only placebo group.

Results of other secondary efficacy analyzes were generally consistent with those of the primary efficacy variable in showing a maintenance of improvement during the open-label in patients initially randomized to opicapone and a rapid increase in improvement followed by maintenance of improvement in patients initially randomized to placebo. The percentage of OFF-time responder (≥ 60 min) was maintained over the open-label period (Table 1). Similarly, the percentage of ON-time responders (≥ 60 min) was maintained over the open-label period (49.4% at open-label baseline to 62.5% at Week 52, Supplementary Table 1). Additional secondary efficacy results related to changes in ON-time, UPDRS, and PDQ-39 for the overall open-label population are summarized in Supplementary Table 2. The effects of opicapone on the UPDRS II (at OFF) and UPDRS III (at ON), indicated that the effect already established during the double-blind phase was maintained during the open-label phase [mean (SD) UPDRS II at OFF: 11.3 (6.9) at open-label baseline to 10.5 (6.2) at Week 52; mean (SD) UPDRS III at ON: 15.2 (9.9) at open-label baseline to 14.1 (9.7) at Week 52]. In terms of non-motor symptoms, there was almost no change in mean UPDRS I from the open-label baseline through to Week 52, similar to the results for the double-blind phase. The percentage of patients with any improvement in CGI-C increased from 57.1% at open-label baseline to 76.2% at Week 52 (Supplementary Table 3). Similarly, the percentage of patients with PGI-C with minimal or greater improvement increased from 48.6% at open-label baseline to 63.4% at Week 52. Finally, the frequency distributions of both the Modified Hoehn and Yahr Stage at ON stage and the Schwab and England ADL Scale Score (at OFF and ON stage) were relatively unchanged between the start and end of the open-label period (Supplementary Fig. 3 and Supplementary Fig. 4).

**Table 1:** OFF-time responders at relevant visits during the open-label period

	Opicapone 50 mg tablets (OL period)			
	All	Placebo (DB period)	Opicapone 25 mg tablets (DB period)	Opicapone 50 mg tablets (DB period)
Week 0 (OL baseline), *N*	385	137	126	122
Responder	187 (48.6)	56 (40.9)	63 (50.0)	68 (55.7)
Non-responder	198 (51.4)	81 (59.1)	63 (50.0)	54 (44.3)
Week 4, *N*	380	133	127	120
Responder	230 (60.5)	81 (60.9)	80 (63.0)	69 (57.5)
Non-responder	150 (39.5)	52 (39.1)	47 (37.0)	51 (42.5)
Week 28, *N*	345	117	116	112
Responder	216 (62.6)	79 (67.5)	69 (59.5)	68 (60.7)
Non-responder	129 (37.4)	38 (32.5)	47 (40.5)	44 (39.3)
Week 52, *N*	315	105	107	103
Responder	188 (59.7)	71 (67.6)	57 (53.3)	60 (58.3)
Non-responder	127 (40.3)	34 (32.4)	50 (46.7)	43 (41.7)

*DB* double-blind, *OL* open-label

### Safety

Results of the safety assessments during the open-label period generally confirmed the tolerability of opicapone noted during the double-blind period.

Of 391 patients, adverse events and adverse reactions (i.e., study drug-related adverse events) were reported by 338 (86.4%) patients and 156 (39.9%) patients, respectively (Table 2). The most common adverse events (incidence of ≥ 3%) were nasopharyngitis, dyskinesia, contusion, constipation, falls, back pain, and weight decrease although these were mild or moderate in severity in most patients (Table 2). Dyskinesia was the most common drug-related adverse event, occurring in 45 (11.5%) patients and similar in incidence (12.4%) to that noted among patients who received opicapone 50 mg tablets in the double-blind period. Of these 45 patients, 31 (68.9%) patients had dyskinesia present at the baseline of the double-blind period. Serious adverse events and drug-related serious adverse events were reported in 57 (14.6%) patients and 10 (2.6%) patients, respectively. Serious adverse events that occurred in ≥ 2 patients included Parkinson’s disease (seven patients), pneumonia aspiration (four patients), pneumonia (three patients), and ileus, spinal compression fracture, benign prostatic hyperplasia, and pleurisy (two patients each) (Table 3). Death due to subdural hematoma was reported in one patient but was not causally related to opicapone administration. Adverse events that resulted in discontinuation were reported in 23 (5.9%) patients whereas 11 (2.8%) patients had study drug-related events that led to discontinuation although no cases of dyskinesia led to study discontinuation.

**Table 2:** Summary of adverse event categories and the frequency of adverse events that occurred in ≥ 3% of patients with the corresponding drug-related adverse event frequency for each event

AE category	Opicapone 50 mg tablets (*n* = 391)	
	AEs, *n* (%)	Drug-related AEs, *n* (%)
Patients with any AEs	338 (86.4)	156 (39.9)
Patients with SAEs	57 (14.6)	10 (2.6)
Patients discontinued due to AEs	23 (5.9)	11 (2.8)
Patients with AEs that resulted in drug withdrawal	8 (2.0)	5 (1.3)
Patients with AEs that resulted in death	1 (0.3)	0 (0.0)

AEs that occurred in ≥ 3% of patients and the corresponding drug-related AE frequency	All AEs, *n* (%)	Drug-related AEs, *n* (%)
All	338 (86.4)	156 (39.9)
Nasopharyngitis	66 (16.9)	1 (0.3)
Dyskinesia	47 (12.0)	45 (11.5)
Contusion	35 (9.0)	7 (1.8)
Constipation	28 (7.2)	14 (3.6)
Fall	25 (6.4)	5 (1.3)
Back pain	23 (5.9)	1 (0.3)
Weight decreased	21 (5.4)	15 (3.8)
Hallucination	17 (4.3)	14 (3.6)
Parkinson’s disease	17 (4.3)	2 (0.5)
Influenza	15 (3.8)	1 (0.3)
Eczema	15 (3.8)	1 (0.3)
Dental caries	12 (3.1)	0 (0.0)

*AE* adverse event, *SAE* serious adverse event

**Table 3:** Serious adverse events that occurred in ≥ 2 patients and drug-related serious adverse events

	Opicapone 50 mg tablets (*N* = 391)
Serious adverse events, *n* (%)	57 (14.6)
Parkinson’s disease	7 (1.8)
Pneumonia aspiration	4 (1.0)
Pneumonia	3 (0.8)
Ileus	2 (0.5)
Spinal compression fracture	2 (0.5)
Benign prostatic hyperplasia	2 (0.5)
Pleurisy	2 (0.5)
Drug-related serious adverse events, *n* (%)	10 (2.6)
Atrioventricular block complete	1 (0.3)
Ileus	1 (0.3)
Volvulus	1 (0.3)
Large intestine polyp	1 (0.3)
Decreased activity	1 (0.3)
Pyrexia	1 (0.3)
Pneumonia	1 (0.3)
Breast cancer	1 (0.3)
Prostate cancer	1 (0.3)
Dyskinesia	1 (0.3)
Parkinson’s disease	1 (0.3)
Hallucination, visual	1 (0.3)

With regard to other safety pre-specified measurements, there were no notable changes over time in the mean quantitative values of hematology, blood biochemistry, urinalysis, cardiovascular, and blood coagulation tests or in physical and neurological examination findings.

Suicidal tendency was reported in 14 (3.6%) patients during the open-label period, of which 13 patients showed only suicidal ideation. At the open-label baseline, 2 of these 14 patients had suicidal ideation and seven patients had anxiety, insomnia or sleep disturbance, which were classified under the system organ class of psychiatric disorders. Suicidal behavior (“suicide attempt” and “aborted attempt”) was noted at Week 28 in one patient but no further attempt was noted at the end of the open-label period. One suicide-related adverse event, which was considered as an adverse reaction, occurred in one (0.3%) patient in the open-label period.

## Discussion

The results of this extension study confirm the long-term safety and efficacy of opicapone tablets at a fixed dose of 50 mg once daily in Japanese patients with PD and motor fluctuations. In particular, the reduction in OFF-time noted during the double-blind period in patients who were randomized to opicapone was maintained for 52 weeks whereas patients who had been in the placebo group in the double-blind period saw an immediate reduction in OFF-time after transfer to open-label opicapone 50 mg followed by a lasting reduction in OFF-time. Similarly, improvements in the change in OFF-time responders were also maintained. Regarding safety, opicapone appeared to be safe and generally well tolerated with long-term treatment despite the use of a fixed-dose schedule similar to that used in real-world settings, of which patients were highly compliant with. Although adverse events during the open-label period were common, there was no marked difference noted in the frequency and severity of adverse reactions compared with those noted with opicapone treatment during the double-blind period. Further, most adverse reactions (i.e., drug-related adverse events) were mild or moderate in severity and the only adverse reaction with a high frequency (incidence > 5%) was dyskinesia, which is a known adverse effect of opicapone. Regarding patients with dyskinesia, most reported this adverse reaction at the initiation of the double-blind period and 14 of 45 (31.1%) patients had a reduction in levodopa dosing over the open-label period, suggesting that some patients may require levodopa dose reduction for dyskinesia control.

The results of this study conducted among Japanese patients are consistent with similar studies in non-Japanese patients, despite key differences in the design of these studies (Ferreira et al. 2018, 2019). In a pooled analysis of the BIPARK-I and BIPARK-II pivotal studies, data from 633 patients who completed the 1-year open-label extension were available (Ferreira et al. 2019). However, in the BIPARK extension studies, a non-fixed-dose schedule was used, in which open-label treatment was started with opicapone 25 mg, which could be titrated to 50 mg if required to control wearing-off and if tolerated. In contrast, the present study used a fixed-dose schedule of opicapone 50 mg tablet over the course of the long-term extension period, which reflects the conditions of real-world clinical practice. In the BIPARK extension studies, patients previously treated with placebo, opicapone 25 mg and opicapone 50 mg during the double-blind phase had additional mean reductions in absolute OFF-time during open-label opicapone administration of − 0.85 h, − 0.32 h and − 0.14 h, respectively (Ferreira et al. 2019). In comparison, Japanese patients in the present study treated with placebo, opicapone 25 mg and opicapone 50 mg during the double-blind phase had additional mean reductions in absolute OFF-time during open-label opicapone administration of − 1.26 h, − 0.37 h and − 0.07 h, respectively. Among the secondary efficacy variables, the pooled analysis of the BIPARK-I and BIPARK-II studies found that the long-term maintenance of clinical effect was confirmed by CGI-C and PGI-C data with 32.9–39.5% and 34.7–40.9% of patients, respectively, rated as being ‘much’ or ‘very much’ improved at the end of the open-label period relative to the double-blind baseline period. In a similar manner, the present study found improvements across the open-label period in CGI-C and, to a slightly lesser extent, in PGI-C. These results broadly conform to those of the present study and confirm the similar efficacy of opicapone in Japanese and non-Japanese patients with PD. The safety and tolerability of opicapone noted in the present study is also consistent with results from previous studies in non-Japanese populations. In particular, there was a lack of hepatic enzyme abnormalities and gastrointestinal problems in studies of both populations that have been noted with other COMT inhibitors (Brooks 2004; Haasio 2010). Instead, dyskinesia appears to be the most common drug-related adverse reaction with opicapone throughout the studies to date.

Limitations of this study include those commonly noted for long-term extension studies. The use of a fixed-dose schedule of opicapone 50 mg tablets can be considered a strength of this study in that it establishes the safety and efficacy of a single dose as may be used over extended periods similar to those in real-world settings. However, this may also represent a limitation in that it is impossible to establish the efficacy and safety of long-term administration of opicapone 25 mg or placebo. However, the general level of agreement in both efficacy and safety findings between Japanese and non-Japanese patients enrolled in similarly designed studies provides reassurance regarding these results.

In conclusion, adjunct opicapone once-daily tablets were generally well tolerated over one year in Japanese L-dopa-treated patients with PD and motor fluctuations. Long-term efficacy in Japanese populations is maintained at similar levels as that obtained during double-blind, placebo-controlled treatment and is also generally consistent with results noted in similar non-Japanese patient populations.

## Supplementary Information

Below is the link to the electronic supplementary material.

Supplementary file1 (DOCX 337 KB)

## Data Availability

Qualified researchers may request ONO Pharmaceutical Co. Ltd. to disclose individual patient-level data from clinical studies through the following website: Clinical Study Data Request.com. For more information on ONO Pharmaceutical Co. Ltd.’s Policy for the Disclosure of Clinical Study Data, please see the following website: https://www.ono.co.jp/eng/rd/policy.html.
