# Supplementary material for: Long-term safety and efficacy of opicapone in Japanese Parkinson’s patients with motor fluctuations
Source: J Neural Transm (Vienna). 2021 Feb 25;128(3):337–44. doi: 10.1007/s00702-021-02315-1 (PMC7969548; doi:10.1007/s00702-021-02315-1)

# Electronic Supplementary Material

**Article title:** Long-term safety and efficacy of opicapone in Japanese Parkinson's patients with motor fluctuations

**Journal:** Journal of Neural Transmission

**Authors**

Atsushi Takeda^1,2^, Ryosuke Takahashi^3^, Yoshio Tsuboi^4^, Masahiro Nomoto^5,6^, Tetsuya Maeda^7^, Akihisa Nishimura^8^, Kazuo Yoshida^8^, Nobutaka Hattori^9^

**Affiliations**

^1^ National Hospital Organization, Sendai-Nishitaga Hospital, Sendai, Japan

^2^ Department of Cognitive & Motor Aging, Tohoku University, Graduate School of Medicine, Sendai, Japan

^3^ Department of Neurology, Kyoto University Graduate School of Medicine, Kyoto, Japan

^4^ Department of Neurology, Fukuoka University Hospital, Fukuoka, Japan

^5^ Department of Neurology and Clinical Pharmacology, Ehime University Graduate School of Medicine, Ehime, Japan

^6^ Department of Neurology, Saiseikai Imabari Hospital, Ehime, Japan

^7^ Division of Neurology and Gerontology, Department of Internal Medicine, School of Medicine, Iwate Medical University, Iwate, Japan

^8^ Department of Clinical Development, Ono Pharmaceutical Co., Ltd., Osaka, Japan

^9^ Department of Neurology, Juntendo University Graduate School of Medicine, Tokyo, Japan

**Corresponding Author:** Atsushi Takeda

**E-mail:** [takeda.atsushi.nc@mail.hosp.go.jp](mailto:takeda.atsushi.nc@mail.hosp.go.jp)

**Supplementary Table 1.** ON-time responders at relevant visits during the open-label period

|  | **Opicapone 50 mg tablets (OL period)** | | | |
| --- | --- | --- | --- | --- |
|  | **All** | **Placebo  (DB period)** | **Opicapone 25 mg tablets  (DB period)** | **Opicapone 50 mg tablets  (DB period)** |
| Week 0 (OL baseline), N | 385 | 137 | 126 | 122 |
| Responder | 190 (49.4) | 58 (42.3) | 63 (50.0) | 69 (56.6) |
| Non-responder | 195 (50.6) | 79 (57.7) | 63 (50.0) | 53 (43.4) |
| Week 4, N | 380 | 133 | 127 | 120 |
| Responder | 213 (56.1) | 71 (53.4) | 76 (59.8) | 66 (55.0) |
| Non-responder | 167 (43.9) | 62 (46.6) | 51 (40.2) | 54 (45.0) |
| Week 28, N | 345 | 117 | 116 | 112 |
| Responder | 204 (59.1) | 73 (62.4) | 68 (58.6) | 63 (56.3) |
| Non-responder | 141 (40.9) | 44 (37.6) | 48 (41.4) | 49 (43.8) |
| Week 52, N | 315 | 105 | 107 | 103 |
| Responder | 197 (62.5) | 72 (68.6) | 61 (57.0) | 64 (62.1) |
| Non-responder | 118 (37.5) | 33 (31.4) | 46 (43.0) | 39 (37.9) |

Abbreviation: DB, double-blind; OL, open-label

**Supplementary Table 2.** Secondary efficacy results presented as change from open-label baseline to Week 52

|  | **Opicapone 50 mg tablets** | |
| --- | --- | --- |
| **Efficacy variable** | **OL baseline** | **Change at Week 52** |
| Change in total ON-time, h; n, mean (SD) | 385 11.29 (3.20) | 313 0.58 (2.40) |
| Change in ON-time without troublesome dyskinesia^a^, h; n, mean (SD) | 385 11.18 (3.16) | 313 0.55 (2.35) |
| Change in ON-time with troublesome dyskinesia, h; n, mean (SD) | 385 0.11 (0.66) | 313 0.03 (0.83) |
| Change in UPDRS I (at ON); n, mean (SD) | 387 0.9 (1.3) | 315 0.0 (1.1) |
| Change in UPDRS II (at OFF); n, mean (SD) | 387 11.3 (6.9) | 315 -0.3 (3.2) |
| Change in UPDRS II (at ON); n, mean (SD) | 387 4.8 (5.0) | 315 -0.1 (2.2) |
| Change in UPDRS III (at ON); n, mean (SD) | 386 15.2 (9.9) | 314 -1.1 (5.3) |
| PDQ-39; n, mean (SD) | 386 19.51 (14.08) | 314 2.84 (9.57) |

Abbreviations: PDQ-39, Parkinson’s Disease Questionnaire; SD, standard deviation; UPDRS, Unified Parkinson’s Disease Rating Scale

The Last Observation Carried Forward (LOCF) method was applied to the handling of missing data

^a^ON-time without troublesome dyskinesia is ON-time without dyskinesia and ON-time with non-troublesome dyskinesia

**Supplementary Table 3.** Frequency distributions of Clinician Global Impression of Change (CGI-C) and Patient Global Impression of Change (CGI-P) at open-label baseline and at Week 52

|  | **CGI-C** | | **CGI-P** | |
| --- | --- | --- | --- | --- |
|  | **OL baseline (N=387)** | **Week 52  (N=315)** | **OL baseline (N=387)** | **Week 52  (N=315)** |
| Very much improved | 14 (3.6) | 25 (7.9) | 8 (2.1) | 13 (4.1) |
| Much improved | 74 (19.1) | 102 (32.4) | 55 (14.2) | 70 (22.2) |
| Minimally improved | 133 (34.4) | 113 (35.9) | 125 (32.3) | 117 (37.1) |
| No change | 144 (37.2) | 65 (20.6) | 140 (36.2) | 80 (25.4) |
| Minimally worse | 20 (5.2) | 10 (3.2) | 49 (12.7) | 27 (8.6) |
| Much worse | 2 (0.5) | 0 (0.0) | 9 (2.3) | 7 (2.2) |
| Very much worse | 0 (0.0) | 0 (0.0) | 0 (0.0) | 1 (0.3) |
| Not assessed | 0 (0.0) | 0 (0.0) | 1 (0.3) | 0 (0.0) |

Abbreviations: CGI-C, Clinician Global Impression of Change; CGI-P, Patient Global Impression of Change; OL, open-label

**Supplementary Fig. 1.** Study design


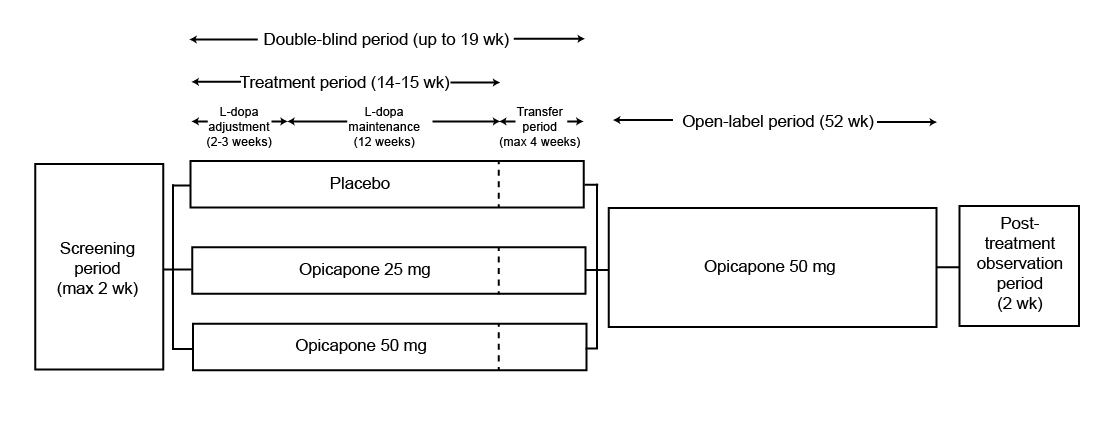


**Supplementary Fig. 2.** Disposition of patients

**
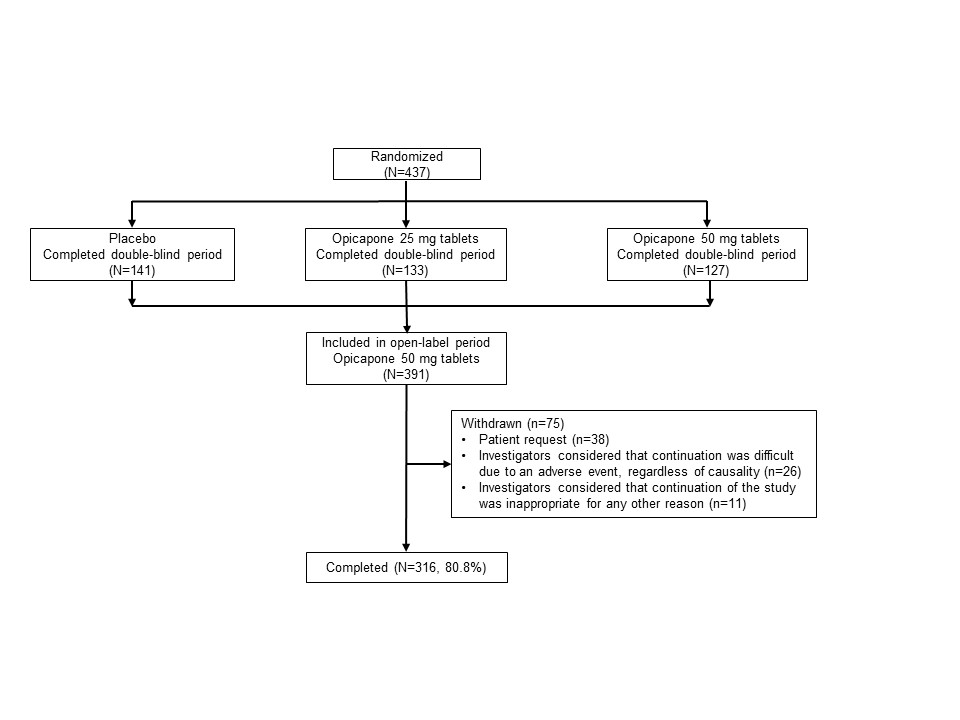
**

**Supplementary Fig. 3.** Frequency distribution of Modified Hoehn & Yahr Stage at ON stage at start (Visit 8) and end (Visit 22) of open-label period.


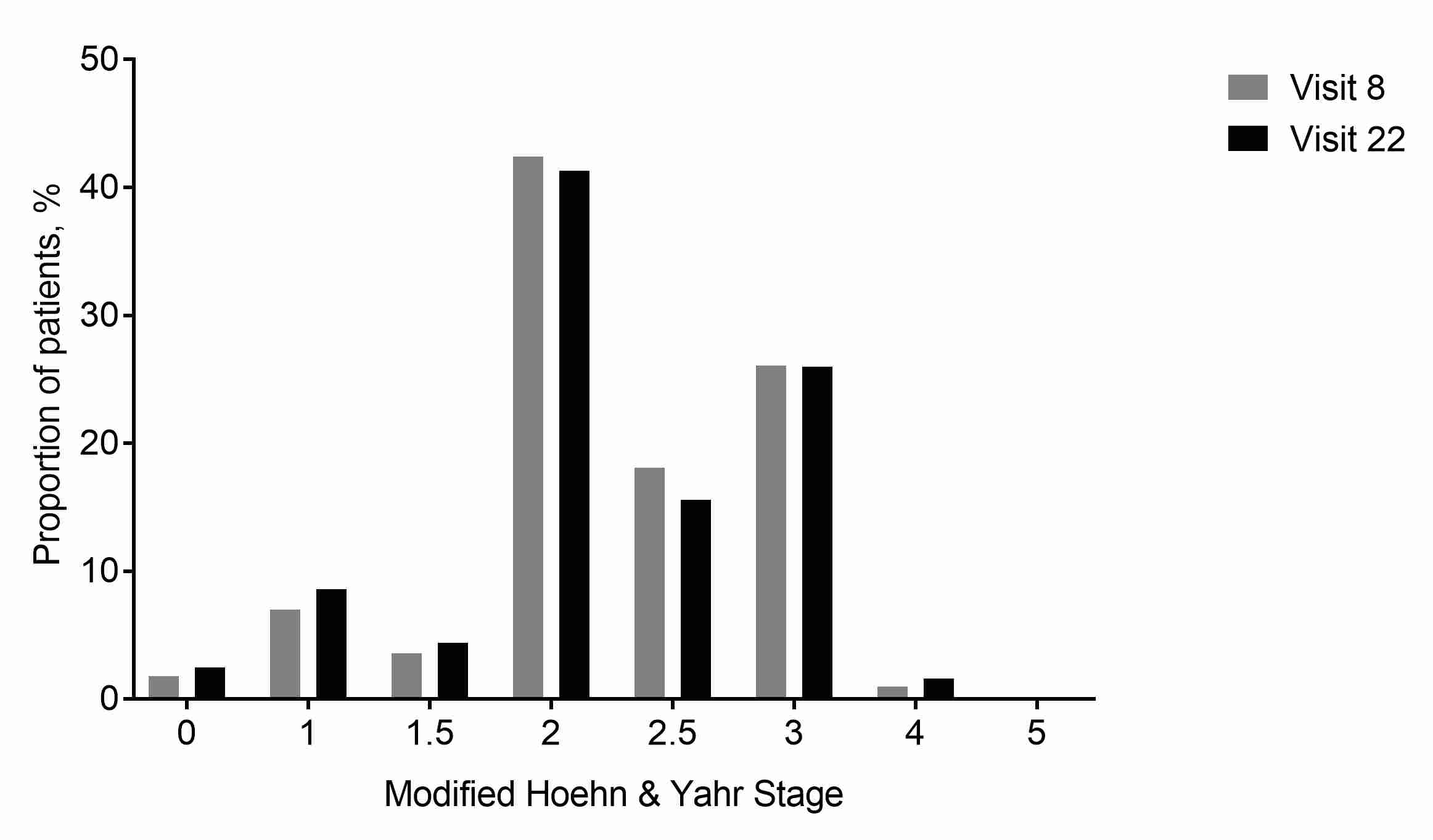


**Supplementary Fig. 4.** Frequency distribution of Schwab and England ADL Scale Score at (A) OFF stage and at (B) ON stage at start (Visit 8) and end (Visit 22) of open-label period.


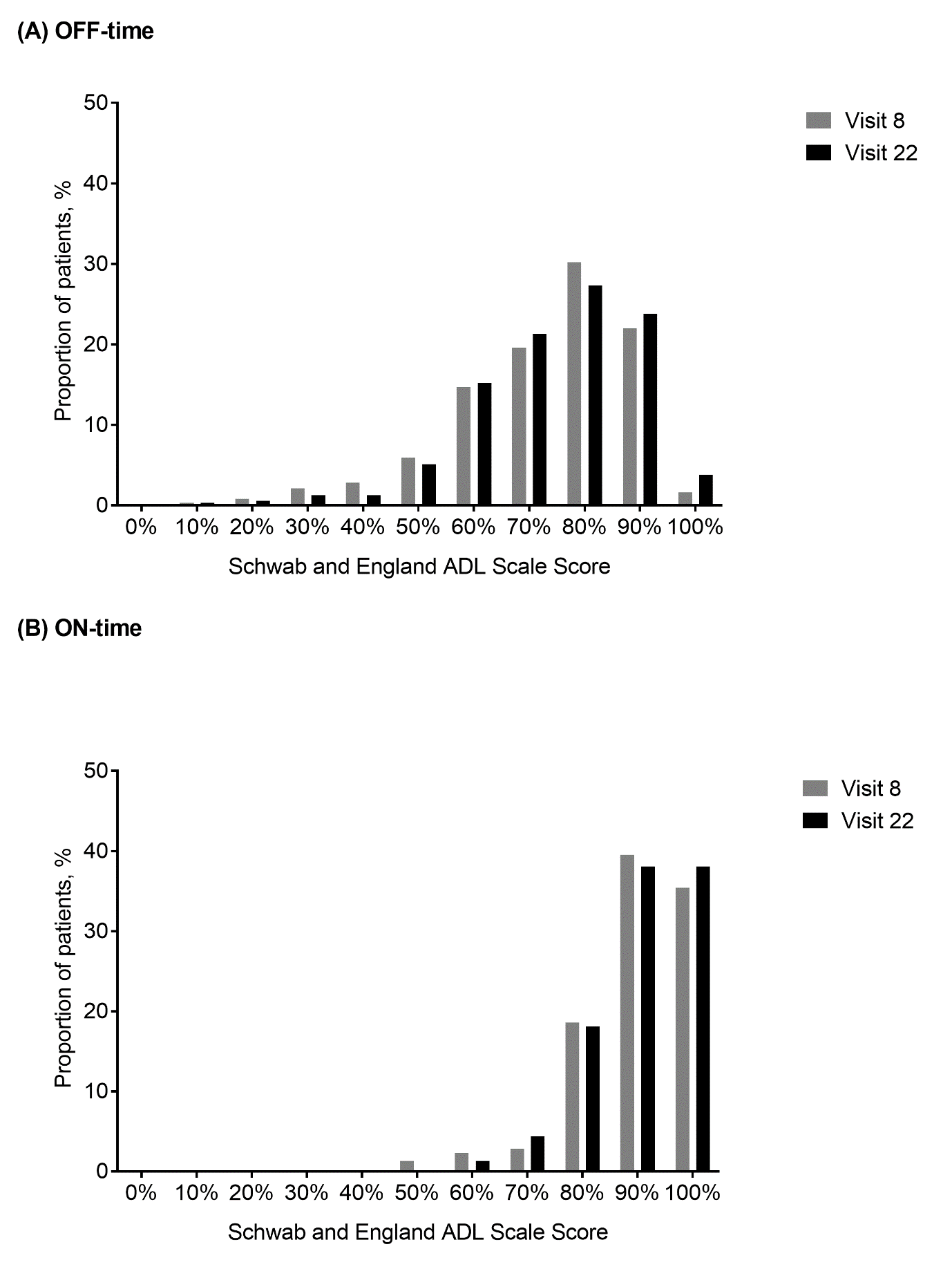

Supplement: Supplementary file 1 — Supplementary file1 (DOCX 337 KB) [file 702_2021_2315_MOESM1_ESM.docx]
